# Retention of health care workers at health facility, trends in the retention of knowledge and correlates at 3rd year following training of health care workers on the prevention of mother-to-child transmission (PMTCT) of HIV–National Assessment

**DOI:** 10.1186/s12913-019-3925-4

**Published:** 2019-01-29

**Authors:** Nyuyki Clement Kufe, Carole Metekoua, Monkam Nelly, Florence Tumasang, Enow Robinson Mbu

**Affiliations:** 1 Medical Research Council (MRC)/Developmental Pathways for Health Research Unit (DPHRU), Department of Paediatrics, School of Clinical Medicine, Faculty of Health Sciences, University of the Witwatersrand, Johannesburg, South Africa; 2 Global Health Metrics (GHM) Research Group, BO Box 8111, Yaoundé, Cameroon; 3 Department of Epidemiology and Biostatistics, School of Public Health, Faculty of Health Sciences, University of the Witwatersrand, Johannesburg, South Africa; 4 Department of Family Health, Ministry of Public Health, Yaoundé, Cameroon

**Keywords:** Retention of knowledge, Prevention of mother-to-child transmission of HIV, Correlates, health care workers

## Abstract

**Background:**

Knowledgeable Health Care Workers (HCWs) are indispensable for the proper management of clients. We investigated retention of HCWs at health facility and retention of knowledge at 18, 24 and 36 months after training and correlates for retention of knowledge at 3rd year.

**Methods:**

A cross-sectional study was conducted among 1000 HCWs, 710 were trained and 290 untrained working at the PMTCT of HIV services in health facilities of the ten regions of Cameroon. A Multiple Choice Questionnaire (MCQ) on HIV management with focus on PMTCT of HIV was used to assess retention of HCWs at the health facility and retention of knowledge. Summary statistics described mean scores for retention of HCWs and retention of knowledge. One-way Analysis of Variance summarized the differences in retention of knowledge over time after training. Correlates for retention of knowledge were investigated by logistic regression analysis.

**Results:**

The retention of HCWs at health facilities in PMTCT of HIV services was 85%. Trained HCWs had higher mean scores for retention of knowledge than untrained HCWs, *p* < 0.001. Knowledge attrition was observed from 18, 24 to 36 months following training. Differences in the mean scores for retention of knowledge were observed between state-owned with private and confessionary health facilities but not among trained HCWs at 18, 24 or 36 months. Highest mean scores for retention of knowledge were observed in District Hospitals, Sub-Divisional Hospitals, and Integrated Health Centres. Correlates for retention of knowledge were: gender, type of health facility, location, longevity at PMTCT services, trained others and had means to apply what was trained to do.

**Conclusion:**

Retention of **t**rained HCWs at health facilities was high, mean scores for retention of knowledge was average and knowledge attrition was observed over time. This research is critical to understand where interventions may be most effective.

## Background

The Millennium Development Goals (MDGs) in most developing countries ended up as unfinished agendas and spilled over to Sustainable Development Goals (SDGs). Three of the MDGs focused on reduction of child and maternal mortality and fighting infectious diseases, to wit: MDG4 (reducing child mortality rates by two-thirds), MDG5 (reducing maternal mortality ratio by three-quarters between 1990 and 2015) and MDG6 (combating HIV/AIDS, malaria and other diseases) [1]. Prevention of Mother-To-Child Transmission (PMTCT) services were developed to address the vertical transmission of Human Immunodeficiency Virus (HIV) from mother to child. The PMTCT of HIV services have witnessed a rapid scale-up due to innovative strategies based on scientific evidence. Significant progress has been made in the global scale-up of PMTCT with a 52% decline in new HIV infections among children between 2001 and 2012 [2]. Globally, in 2016, about 160,000 new paediatric infections were recorded as against 300,000 in 2010 and more than 900,000 pregnant women living with HIV accessed PMTCT services (62% coverage) [3, 4]. In Sub-Saharan Africa, challenges in the implementation of PMTCT of HIV included; the poor adherence to guidelines and recommendations by HCWs or HIV-infected women, inadequacies in the full integration of HIV testing and treatment into reproductive health services and insufficient skilled HCWs [5].

The healthcare sector unlike others is highly specialized. The clients to the healthcare industry rely heavily upon the highly skilled personnel who must be sufficiently knowledgeable and competent. The talent pool in the health care industry is not easily available and takes time to build. Trained individuals may forget important skills which lead to decreased performance and knowledge attrition over a period of time [6]. Knowledge is a vital source of competitive advantage though relatively neglected [7]. To maintain a skilled, knowledgeable and competent workforce targeted training is used as the primary approach to impart and upgrade skills and assure retention of knowledge. The loss of a single effective and knowledgeable person can disrupt the sane functioning of a unit or service. Studies have shown positive outcomes after training though some have not. Training of HCWs improves care provision and reduction in neonatal mortality [8, 9]. Improvement in service delivery at PMTCT services reduces mother to child transmission of HIV to less than 5%, and timely provision of packages of evidence-based interventions leads to reduction of maternal and new-born deaths [10]. To ensure better outputs of frontline HCWs capacity building through targeted training and task shifting is implemented permitting better integration of HCWs into the care system [11].

Cameroon committed itself to scale-up PMTCT program with a goal of reaching 90% of pregnant women with PMTCT services by 2015 [12]. The PMTCT program initiated in 2000 developed policies and guidelines. A minimum package of PMTCT services were provided in health facilities be they public, private, Faith-based or Non-Governmental Organization (NGO) owned. The Presidents’ Emergency Plan for AIDS Relief (PEPFAR) funded PMTCT of HIV in the Department of Family Health (DFH) of the Ministry of Health (MoH) Cameroon with a component to update the knowledge, competences and skills of HCWs on various aspects of PMTCT such as Antenatal Care (ANC), Anti-Retroviral (ARV) management, and referrals as prescribed by World Health Organisation (WHO) by training health care providers working at the health facilities of the ten regions of the country. Between 2011 and 2015, HCWs working at the PMTCT of HIV, Maternal, Neonatal and Child Health (MNCH) and Paediatric HIV services were trained by the PEPFAR team of the DFH, MoH on various aspects of Sexual and Reproductive Health (SRH) including service integration, emergency obstetrics and neonatal care (EmONC), family planning (FP) and PMTCT of HIV. An assessment in one region described the available equipment/material, health care provider’s knowledge before training and retention of knowledge after 7 months showed improved competence of nurses on PMTCT following a short training [13]. Research findings on HIV/AIDS are paltry from central and western African countries. Studies on motivation and retention of HCWs have been carried out in African countries [14, 15] but non on the retention of HCWs on specific services and on retention of knowledge and clinical application of the PMTCT of HIV content over a period of time. Training considerably improves healthcare provider’s skills and knowledge. However, follow-up evaluations have shown a gradual decline from post-training scores though the knowledge stayed significantly above baseline over time [14, 15]. In line with key research priorities for accurate monitoring of progress and challenges, informing policy and effective program planning from evidenced informed disaggregated data [16] we investigated the retention of HCWs in PMTCT of HIV/AIDS, MNCH and Paediatric services, the retention of knowledge by the trained HCWs at 18, 24 and 36 months after training and correlates of retention of knowledge at 3rd year after training.

## Methods

### Study population

Between January 2011 and June 2015 about 1720 health providers underwent training in all the ten regions of Cameroon on family planning, PMTCT of HIV, integration of PMTCT into district health services (DHS), EmONC, PMTCT/Option B+. The study population consisted of HCWs trained by the PEPFAR team on PMTCT of HIV referred to as trained HCWs and HCWs not trained by the PEPFAR team referred to as untrained HCWs. The Health Care Workers included: medical doctors (general practitioners (GPs) and specialists-paediatricians, gynaecologists), midwives, nurses, nurse-aids and medical technicians working at PMTCT of HIV, MNCH and Paediatric HIV services for at least 1 year from state-owned, private and Faith-Based health facilities.

### Health facilities

Level 1 (General Hospitals), Level 2 (Central Hospitals) and Level 3 (Regional Hospitals) and hospitals classified as such, district hospitals (DHs), sub-divisional hospitals (SDHs), integrated health centres (IHCs) and health centres (HCs) of the ten regions constituted the study sites. Criteria for selection of health facilities were based on the existence of PMTCT of HIV services and high turnovers.

### Sampling and study design

A purposeful sample of HCWs was drawn from PMTCT of HIV, MNCH and Paediatric services in health facilities throughout the country from 30th May 2016 to 30th June 2016. In each health facility, for every three HCWs trained on the PMTCT of HIV an untrained HCW working at PMTCT of HIV, MNCH and Paediatric services and carrying out similar functions was also invited to take part in the assessment. The study design was cross sectional.

### Procedure

#### Assessment of health care workers

Two different instruments assessed knowledge and skills of HCWs individually and in isolation. An appointment was taken at the health facility of the HCW depending on the schedule for the week. Consented HCWs working at either the PMTCT of HIV, MNCH or Paediatric services for at least a year responded to two anonymous bilingual (English and French) questionnaires in the language of their preference. First part of the assessment was an interviewer administered questionnaire used for the first part of the knowledge as well as skill assessment. The questionnaire was divided into section A (identification: region, health district, name, type and category of health facility, zone of location: rural/urban, trained by PEPFAR team on PMTCT of HIV within the past 18 to 36 months, If Yes, how many months ago? 18 months to less than 24 months, ≥24 months but < 36 months, ≥36 months, received training hand-outs during training from PEPFAR team?), section B (socio-demographic data and unit/service of work, services offered and frequency (daily, weekly or monthly), longevity as a health care provider, currently working on PMTCT and how many years of work on PMTCT services, had been supervised by PEPFAR personnel on PMTCT of HIV activities after training), section C (work specifications: outpatient and antenatal consultation, family planning, labour and delivery, postpartum, post natal consultations, paediatric consultations, follow up of HIV+ mothers up to 24 months, surveillance of ARV side effects, and vaccinations) and section D (important aspects retained during the training on PMTCT of HIV, difficult aspects during training, trained other colleagues onsite on PMTCT of HIV, and whether the HCW had the means to apply what s/he was trained to do and whether there exist a conducive environment to work on PMTCT of HIV). Second part of the assessment differed for knowledge and skill evaluation. For knowledge assessment, the questionnaire was a self-administered multiple choice questionnaire (MCQ) in the presence of the interviewer and it consisted of 30 questions on general as well as factual information on PMTCT of HIV selected from pre- and post-tests used during the training of HCWs by the PEPFAR team but in a different order. HCWs responded in the health facility in writing for 30 to 50 min. Neither the answers nor the scores of the tests were revealed to the participants. For skills assessment, a checklist on provider initiated counselling and testing, breast feeding in the event of PMTCT of HIV and enhanced delivery techniques was used to observe participants at work and verify records and documents comprising ten aspects; namely, antenatal consultation, delivery, post-natal care for the child, management and ART for the child, post-natal care for the mother, family planning/HIV integration, management of PMTCT/MNCH commodities, waste management, management of needle prick/exposure to biological fluids and management of PMTCT/MNCH programme at the site.

### Data management and statistical analysis

A sample size of 1000 was sufficient to detect an effect size (Cohen f^2^) of 0.02 for a multivariable model comprising of 10 covariates to explain group variance and allow for separate models for trained and untrained HCWs in quantitative analysis. Data was captured with Epi Data 3.0 and analysed using STATA 14.2 SE (StataCorp.2012. College Station, TX: StataCorp LP). Data was weighted, adjusted and computed within 95% Confidence Intervals (CIs) and at significance of *p* < 0.05. Summary statistics are presented as percentages (%), mean scores (x̅) and standard deviations (SD) for all continuous variables. Chi-square tests for categorical variables, independent Student’s T tests for continuous variables and One-way Analysis of Variance (ANOVA) assessed the differences at 18, 24 and 36 months. Post-hoc Tukey tests were implemented to identify the variables involved. A score ≥ 50% was adopted for retention of knowledge. We investigated the relationship between retention of knowledge and covariates by logistic regression. To investigate the factors responsible for retention of knowledge we carried out multivariable logistic regression analysis by backward selection in an unadjusted model with all significant (*p* < 0.05) factors in the univariate analysis and adjusted model for region. Model assumptions were checked and variables were checked for collinearity and correlation.

## Results

### Descriptive results

A total of 1280 HCWs responded to the questionnaires, 1033 HCWs to retention of knowledge and 247 HCWs responded to the skills questionnaire. We analysed data for retention of knowledge for 1000 HCWs from 72 out of 174 (41.4%) health districts in the ten regions of Cameroon [17]. A total of 71% of the HCWs were trained on PMTCT of HIV and 75% were females. The mean age of the participants was 37.1 ± SD:8.9, men 38.8 ± SD:8.8 and women 36.5 ± SD:8.9, *p* = 0.0004. Trained participants were older than untrained, mean age for trained HCWs was 38.7 ± SD:8.8 and for untrained 33.2 ± SD:7.9, *p* < 0.0001. The mean longevity in service of HCWs was 11.1 ± SD:8.6 years, trained 12.6 ± SD:8.6 years as compared to 7.5 ± SD:6.6 years for the untrained, p < 0.0001. Majority (37.9%) of the participants were aged 30–39 years, married/cohabitating (58.8%), were from integrated/health centres (47.8%), from public health facilities (64.0%), a quarter were nurse-aids, mostly from urban areas (63.0%) and were permanent workers (78%). Table 1 gives details of socio-demographic characteristics.

**Table 1:** Socio-demographics of participants

Variables, n (%)	All, 1000 (100%)	Untrained, 290 (29%)	Trained, 710 (71%)	*p*-value
Gender				
Male	253 (25.3)	59 (20.3)	194 (27.3)	
Female	747 (74.7)	231 (79.7)	516 (72.7)	0.021
Marital status				
Married/Cohabitating	588 (58.8)	152 (52.4)	436 (61.4)	
Single	368 (36.8)	130 (44.8)	238 (33.5)	
Divorced	10 (1.0)	2 (0.7)	8 (1.1)	
Widow (er)	34 (3.4)	6 (2.1)	28 (3.9)	0.006
Age category				
20–29	229 (22.9)	115 (39.7)	114 (16.1)	
30–39	379 (37.9)	114 (39.3)	265 (37.3)	
40–49	279 (27.9)	48 (16.6)	231 (32.5)	
≥50	113 (11.3)	13 (4.5)	100 (14.1)	< 0.001
Type of health facility				
Public	640 (64.0)	201 (69.3)	439 (61.8)	
Private	200 (20.0)	56 (19.3)	144 (20.3)	
Faith-Based	160 (16.0)	33 (11.4)	127 (17.9)	0.025
Category of health facility				
Level 1 (High)	111 (11.1)	28 (9.7)	83 (11.7)	
Level 2 (Medium)	411 (41.1)	130 (44.8)	281 (39.6)	
Level 3 (Lowest)	478 (47.8)	132 (45.5)	346 (48.7)	0.273
Employment status				
Permanent	780 (78.0)	178 (61.4)	602 (84.8)	
On internship	93 (9.3)	51 (17.6)	42 (5.9)	
Temporary	127 (12.7)	61 (21.0)	66 (9.3)	< 0.001
Grade				
Specialists	184 (18.4)	47 (16.2)	137 (19.3)	
General Practitioners (GP)	54 (5.4)	18 (6.2)	36 (5.1)	
Registered nurses	209 (20.9)	45 (15.5)	164 (23.1)	
Midwives	157 (15.7)	31 (10.7)	126 (17.7)	
Nurse-aids	249 (24.9)	103 (35.5)	146 (20.6)	
Medical technicians	147 (14.7)	46 (15.9)	101 (14.2)	< 0.001
Location				
Urban	630 (63.0)	177 (61.0)	453 (63.8)	
Semi-urban	185 (18.5)	61 (21.0)	124 (17.5)	
Rural	185 (18.5)	52 (17.9)	133 (18.7)	0.419
Years of work				
1–9 years	526 (52.6)	212 (73.1)	314 (44.2)	
10–19 years	323 (32.3)	61 (21.0)	262 (36.9)	
≥20 years	151 (15.1)	17 (5.9)	134 (18.9)	< 0.001
Longevity at PMTCT				
0 years	207 (20.7)	102 (35.2)	105 (14.8)	
1–9 years	150 (15.0)	64 (22.1)	86 (12.1)	
10–19 years	156 (15.6)	34 (11.7)	122 (17.2)	
≥20 years	487 (48.7)	90 (31.0)	397 (55.9)	< 0.001
Level of education				
Elementary	34 (3.4)	9 (3.1)	25 (3.5)	
Secondary	229 (22.9)	65 (22.4)	164 (23.1)	
High school	466 (46.6)	130 (44.8)	336 (47.3)	
University/Postgraduate	271 (27.1)	86 (29.7)	185 (26.1)	0.704
Trained others?				
No	424 (42.4)	284 (97.9)	140 (19.7)	
Yes	576 (57.6)	6 (2.1)	570 (80.3)	< 0.001
Have the means to apply what you were trained to do?				
No	391 (39.1)	239 (82.4)	152 (21.4)	
Yes	609 (60.9)	51 (17.6)	558 (78.6)	< 0.001
Conducive environment to carry out PMTCT of HIV?				
No	371 (37.1)	158 (54.5)	213 (30.0)	
Yes	629 (62.9)	132 (45.5)	497 (70.0)	< 0.001
Received training hand-outs?				
No	469 (46.9)	280 (96.6)	189 (26.6)	
Yes	531 (53.1)	10 (3.4)	521 (73.4)	< 0.001
Working in PMTCT services				
No	201 (20.1)	94 (32.4)	107 (15.1)	
Yes	799 (79.9)	196 (67.6)	603 (84.9)	< 0.001

A general retention rate of 84.9% was observed for the trained HCWs at PMTCT of HIV services with little variation from 18 months (85.7%), 24 months (84.6%) and at 36 months (84.5%). Generally, the mean score for retention of knowledge for the 1000 HCWs was 50.7 ± 16.6 (CI:49.7–51.8), for trained HCWs: 53.6 ± 15.9 (CI:52.4–54.8) was average but higher than for the untrained HCWs: 43.7 ± 16.2 (CI:41.9–45.6), which was most often below average. The difference in the mean score of trained and untrained score was statistically significant, *p* < 0.0001. Among the trained HCWs a progressive knowledge retention attrition was observed from 18 months, through 24 months to 36 months but with no statistically significant differences (*p* = 0.1155, Bonferroni) in the mean scores of knowledge retention attrition.

Our results indicate that for the trained HCWs: 67.9% at 18 months, 64% at 24 months and 58.3% at 36 months had mean scores for retention of knowledge of 54.8, 53.9 and 51.7% respectively as compared to 41.4% of the untrained HCWs with mean score of 43.7%.

For trained HCWs, statistically significant differences were observed between the mean scores of male and female HCWs, marital status, type and category of health facility, employment status of HCWs, grade, location, longevity at PMTCT, level of education, trained others, had means to apply what was trained to do, received training handouts, and presently working in PMTCT services. After Post-Hoc testing the groups responsible for the differences among the trained HCWs were as indicated in Table 2. No statistically significant differences were observed for trained HCWs at 18 months, 24 months and 36 months for age category, years of work and whether or not in a conducive environment for PMTCT of HIV, Table 3.

**Table 2:** Distribution of mean scores for retention of knowledge, p-value for trained participants

Variable	All	Untrained	18 months^a^	24 months^b^	36 months^c^	*p*-value
n (%)	1000	290 (29.0)	237 (23.7)	267 (26.7)	206 (20.6)	
Number of participants with a mean score ≤ 50, n (%)	572 (57.2)	120 (41.4)	161 (67.9)	171 (64.0)	120 (58.3)	< 0.0001
Mean score, x̅ (SD)	50.7 (16.6)	43.7 (16.2)	54.8 (16.3)	53.9 (15.7)	51.7 (15.6)	0.0001^d^
Gender						
Male	54.7 (16.1)	46.9 (15.7)	58.7 (14.9)	59.9 (15.1)	52.1 (15.3)	
Female	49.4 (16.6)	42.9 (16.3)	53.7 (16.5)	51.5 (15.3)	51.6 (15.8)	0.0001^e^
Marital status						
Married/Cohabitating	52.0 (16.0)	43.2 (15.3)	55.9 (15.8)	55.5 (14.9)	53.5 (14.3)	
Single	48.9 (17.3)	44.7 (17.0)	52.9 (17.3)	51.2 (16.4)	48.9 (17.6)	
Divorced	43.0 (24.0)	20.0 (28.3)	60.0 (−-)	45.0 (23.4)	60.0 (−-)	
Widow (er)	51.3 (14.5)	45.0 (14.9)	54.0 (13.2)	55.6 (13.6)	46.3 (15.8)	0.0171
Age category						
20–29	48.4 (16.9)	43.7 (17.3)	56.9 (13.4)	47.6 (13.1)	54.6 (21.5)	
30–39	51.3 (16.4)	45.3 (15.6)	54.7 (17.2)	55.4 (15.3)	51.2 (15.5)	
40–49	52.1 (17.4)	41.4 (15.1)	55.1 (17.3)	55.3 (17.8)	52.4 (15.7)	
≥50	50.2 (14.4)	38.9 (15.4)	48.7 (15.3)	54.1 (13.3)	50.5 (13.0)	0.5360
Type of HF						
Public	52.2 (17.1)	45.1 (16.6)	55.9 (17.3)	57.0 (15.8)	53.1 (15.9)	
Private	48.0 (16.0)	40.2 (14.9)	55.7 (14.5)	48.5 (16.4)	47.5 (13.9)	
Faith-Based	48.4 (14.5)	41.4 (15.3)	49.5 (14.1)	50.7 (12.4)	50.3 (15.8)	0.0004
Category of HF						
Level 1 (High)	47.7 (17.3)	41.4 (16.1)	51.5 (18.2)	50.7 (17.2)	47.9 (16.9)	
Level 2 (Medium)	52.7 (17.4)	45.2 (17.6)	58.2 (16.9)	56.5 (16.7)	53.5 (14.4)	
Level 3 (lowest)	49.8 (15.6)	42.8 (14.8)	53.0 (15.0)	52.5 (14.4)	51.5 (16.1)	0.0009
Employment status						
Permanent	52.1 (16.4)	44.7 (16.1)	55.6 (16.1)	54.9 (15.4)	52.2 (15.8)	
Intern	45.7 (17.6)	40.0 (16.3)	53.0 (17.5)	54.2 (15.6)	47.8 (18.9)	
Temporary	46.0 (15.7)	44.2 (16.3)	49.8 (16.3)	44.7 (15.5)	49.0 (13.3)	0.0055
Grade						
Specialists	51.9 (15.6)	47.1 (15.3)	52.4 (15.7)	53.6 (15.6)	55.0 (15.3)	
General Practitioners (GP)	50.9 (18.5)	40.2 (15.2)	58.7 (20.3)	59.8 (15.4)	40.6 (11.6)	
Registered nurses	54.9 (14.8)	48.0 (11.0)	59.7 (14.9)	56.7 (15.4)	54.4 (14.8)	
Midwives	53.5 (17.2)	51.6 (19.4)	53.9 (18.4)	54.1 (16.3)	53.9 (16.1)	
Nurse-aids	46.7 (16.5)	39.7 (16.4)	52.4 (15.9)	50.9 (14.0)	51.3 (14.5)	
Medical technicians	47.2 (16.9)	41.3 (16.4)	55.2 (15.3)	48.9 (16.6)	42.9 (16.1)	0.0071
Location						
Urban	50.1 (16.6)	42.8 (15.6)	54.2 (16.8)	53.4 (16.3)	50.7 (15.2)	
Semi-urban	49.5 (17.1)	42.7 (18.2)	54.8 (14.0)	53.7 (14.1)	49.8 (18.3)	
Rural	54.3 (15.6)	48.4 (15.5)	56.4 (16.4)	57.3 (14.7)	56.4 (13.8)	0.0500
Years of work						
1–9 years	49.4 (17.1)	43.7 (16.4)	54.5 (15.5)	53.3 (16.6)	50.7 (18.3)	
10–19 years	51.9 (16.2)	44.5 (16.5)	56.0 (17.8)	53.1 (15.0)	52.1 (14.6)	
≥20 years	52.9 (15.2)	41.8 (13.0)	53.4 (16.5)	57.1 (14.5)	52.4 (14.1)	0.8026
Longevity at PMTCT						
0 years	45.8 (18.1)	42.5 (17.5)	54.1 (18.0)	47.8 (18.5)	45.9 (17.7)	
1–9 years	51.6 (16.8)	44.5 (16.4)	55.7 (13.6)	57.4 (16.8)	58.5 (15.8)	
10–19 years	51.6 (15.5)	41.6 (15.1)	54.5 (15.7)	54.1 (10.6)	53.9 (12.9)	
≥ 20 years	52.3 (15.8)	45.4 (15.0)	54.9 (17.4)	54.6 (15.1)	52.2 (14.9)	0.0039
Level of education						
Elementary	48.9 (18.6)	45.5 (16.9)	45.4 (25.1)	56.7 (15.9)	49.5 (12.1)	
Secondary	46.5 (16.2)	39.7 (15.9)	49.7 (14.3)	48.8 (16.5)	49.1 (15.6)	
High school	51.1 (16.2)	43.2 (17.2)	55.2 (15.3)	54.5 (14.1)	52.5 (14.8)	
University/PG	54.0 (16.7)	47.4 (14.3)	59.4 (16.4)	57.4 (16.5)	53.2 (17.9)	< 0.0001
Trained others?						
No	45.3 (15.9)	43.7 (16.3)	53.2 (15.2)	47.8 (13.9)	46.0 (14.9)	
Yes	54.8 (15.9)	47.2 (11.4)	55.1 (16.5)	55.5 (15.8)	53.6 (15.5)	0.0001
Have the means to apply what you were trained to do?						
No	46.1 (16.9)	43.3 (16.9)	51.8 (16.7)	50.8 (15.4)	49.1 (16.2)	
Yes	53.7 (15.7)	45.7 (12.9)	55.5 (16.2)	54.9 (15.6)	52.6 (15.4)	0.0073
Conducive environment to carry out PMTCT of HIV?						
No	49.2 (17.0)	43.5 (16.6)	53.9 (17.7)	54.8 (14.9)	51.2 (15.9)	
Yes	51.7 (16.3)	44.0 (15.8)	55.1 (15.8)	53.5 (16.0)	52.1 (15.5)	0.8191
Received training handouts?						
No	46.9 (16.8)	43.7 (16.4)	54.6 (17.6)	49.3 (16.5)	51.3 (13.7)	
Yes	54.1 (15.7)	45.3 (8.3)	54.9 (15.9)	55.5 (15.1)	51.9 (16.4)	0.0512
Working in PMTCT services						
No	46.3 (17.5)	43.1 (16.8)	52.3 (17.3)	49.4 (17.5)	45.3 (18.0)	
Yes	51.9 (16.2)	44.0 (15.9)	55.3 (16.1)	54.8 (15.2)	52.9 (14.9)	0.0013

^a^18 months: ≥18 months but less than 24 months

^b^24 months: ≥24 months but less than 36 months

^c^36 months: ≥36 months

^d^ANOVA

^e^T test

**Table 3:** Pairwise statistically significant differences for retention of knowledge after Post Hoc tests

Variable	Group	Contrast	Tukey p-value	Tukey 95% CI
Marital status	Married vs single	−3.9	0.012	−7.2 to −0.6
Type of HF	Private vs Public	−4.4	0.010	−7.9 to −0.8
	Faith-based vs Public	−5.2	0.003	−8.9 to −1.5
Category of HF	Level 2 vs Level 1	6.4	0.004	1.7–11.0
	Level 3 vs level 1	− 3.7	0.009	−6.7 to − 0.8
Employment status	Temporary vs Permanent	−6.6	0.004	−11.4 to − 1.8
Grade	Nurse-aids vs Registered Nurses	−5.3	0.040	−10.4 to −0.1
	Medical technicians vs Registered nurses	−6.9	0.007	−12.7 to − 1.3
Zone	Rural vs Urban	3.7	0.046	0.05–7.4
Level of education	High school vs secondary school	4.9	0.005	1.1–8.8
	University/PG vs secondary school	7.9	0.000	3.6–12.3

Married/cohabitating, aged 20–29 years, males HCWs, being from public and from level 2 (Medium) health facilities, permanent worker, specialists and registered nurses, from rural areas, older, having a longevity of 1–9 years, having attained a higher level of education, trained others, and presently working in PMTCT services had higher mean scores than other counterparts.

There was a slight improvement in knowledge retention from 18 months to 24 months for trained HCWs who had a conducive environment to implement PMTCT of HIV but a deterioration in knowledge retention was observed for trained HCWs who trained others and also for those who were given the means to practice what they were trained on.

### Correlates of knowledge retention

#### Univariate analysis

Statistically significant covariates included: category and type of health facility, location, gender, employment status, marital status, longevity in PMTCT services, HCWs who trained others, HCWs who had the means to practice what there were trained to do, and working at PMTCT services. The following variables were not statistically significant; age, grade, years of work, level of education, conducive environment to carry out PMTCT of HIV and received training handouts.

#### Multivariable logistic regression

The covariates that maintained statistical significance in multivariable logistic regression model adjusted for region were gender (female: OR = 0.66, CI:0.44–0.98, *p* = 0.044), type of health facility (Faith-based: OR = 0.54, CI:0.34–0.86, *p* = 0.009), employment status (Temporary: OR = 0.47, CI:0.26–0.84, *p* = 0.011), location (Rural: OR = 1.69, CI:1.00–2.88, *p* = 0.049), had the means to practice what they were trained (Yes: OR = 1.55, CI:1.02–2.35, *p* = 0.040) and moderate statistical significance for HCWs who trained others (Yes: OR = 1.55, 1.00–2.39, *p* = 0.050), (Table 4).

**Table 4:** Univariate and multivariable logistic regression analysis for knowledge retention for trained participants, (*n* = 710)

	Univariate logistic analysis			Multivariable logistic analysis					
Factors	OR	95% CI	*p*-value	UOR	95% CI	*p*-value	AOR	95% CI	*p*-value
Gender									
Male	Ref			Ref			Ref		
Female	0.61	0.43–0.87	0.007	0.60	0.41–0.89	0.012	0.66	0.44–0.98	0.044
Marital status									
Married/Cohabitating	Ref			Ref			Ref		
Single	0.69	0.49–0.96	0.027	0.90	0.63–1.29	0.578	0.86	0.58–1.26	0.436
Divorced	1.48	0.29–7.42	0.634	3.15	0.55–17.93	0.197	4.49	0.77–26.21	0.095
Widow (er)	0.57	0.26–1.23	0.151	0.63	0.28–1.43	0.266	0.67	0.29–1.56	0.353
Age category									
20–29	Ref								
30–39	0.97	0.61–1.53	0.887						
40–49	1.06	0.66–1.69	0.808						
≥50	0.66	0.38–1.15	0.140						
Type of health facility									
Public	Ref			Ref			Ref		
Private	0.57	0.39–0.84	0.005	0.78	0.50–1.21	0.261	0.82	0.51–1.32	0.408
Confessionary or Faith based	0.58	0.39–0.87	0.009	0.61	0.39–0.94	0.026	0.54	0.34–0.86	0.009
Category of health facility									
Level 1 (High)	Ref			Ref			Ref		
Level 2 (Medium)	2.08	1.26–3.43	0.004	1.72	1.01–2.93	0.047	1.47	0.83–2.62	0.189
Level 3 (Lowest)	1.39	0.86–2.24	0.185	1.09	0.63–1.87	0.761	1.13	0.63–1.99	0.688
Employment status									
Permanent	Ref			Ref			Ref		
On internship	0.86	0.45–1.63	0.641	0.92	0.45–189	0.822	0.88	0.42–1.85	0.732
Temporary	0.49	0.29–0.83	0.007	0.51	0.29–0.90	0.020	0.47	0.26–0.84	0.011
Grade									
Specialists	Ref								
General Practitioner (GP)	1.36	0.59–2.88	0.507						
Registered nurses	1.43	0.88–2.32	0.148						
Midwives	0.87	0.59–1.44	0.595						
Nurse-aids	0.89	0.55–1.45	0.659						
Medical technicians	0.78	0.46–1.31	0.343						
Location									
Urban	Ref			Ref			Ref		
Semi-urban	0.93	0.62–1.39	0.732	0.97	0.63–1.51	0.923	1.04	0.66–1.65	0.862
Rural	1.91	1.23–2.95	0.004	2.01	1.24–3.26	0.005	1.69	1.00–2.88	0.049
Years of work									
1–9 years	Ref								
10–19 years	1.06	0.75–1.49	0.732						
≥20 years	1.09	0.72–1.67	0.660						
Longevity at PMTCT services									
0 years	Ref			Ref			Ref		
1–9 years	4.03	2.13–7.62	0.000	2.75	1.09–6.89	0.031	2.23	0.85–5.81	0.101
10–19 years	2.34	1.37–4.01	0.002	1.66	0.71–3.89	0.244	1.70	0.69–4.15	0.242
≥ 20 years	2.03	1.31–3.14	0.001	1.27	0.57–2.81	0.562	1.13	0.47–2.59	0.773
Level of education									
Elementary	Ref								
Secondary	0.85	0.36–2.00	0.714						
High school	1.20	0.52–2.75	0.667						
University/PG	1.54	0.65–3.63	0.328						
Trained others?									
No	Ref			Ref			*Ref*		
Yes	1.89	1.29–2.75	0.001	1.71	1.13–2.59	0.011	*1.55*	*1.00–2.40*	*0.050*
Had the means to apply what you were trained to do?									
No	Ref			Ref			Ref		
Yes	1.62	1.13–2.34	0.009	1.46	0.98–2.18	0.064	1.55	1.02–2.35	0.040
Conducive environment to carry out PMTCT of HIV?									
No	Ref								
Yes	1.11	0.79–1.55	0.540						
Received training handouts?									
No	Ref								
Yes	1.25	0.89–1.76	0.197						
Presently working at PMTCT services									
No	Ref			Ref			Ref		
Yes	2.18	1.44–3.30	< 0.001	1.69	0.77–3.70	0.190	1.67	0.74–3.78	0.216

## Discussion

There are several challenges to the scale-up of PMTCT of HIV. The most important and which can be addressed is the frequent changes of staff or transfer of personnel [18]. We observed a high retention of HCWs in PMTCT of HIV services over a period of 3 years.

We ascertained knowledge attrition over a 3 year period regardless of covariate (Table 2). This is in-line with another study which observed a decrease to less than 50 % of knowledge in the second year [19] though our study projected such a decrease in the 4th year. Another assessment on the Integrated Management of Neonatal and Childhood Illness [20] described a significant decrease in knowledge after 3 years. However, another assessment after implementation of the Integrated Management of Childhood Illness strategy showed no waning in the performance of HCWs [21]. This is likely due to methodological approaches and outcomes in the assessment.

Our study observed that training enhances knowledge retention. This is corroborated by other studies which showed that training improved health worker performance with considerable training benefits to HCWs especially in resource limited settings like ours underpinned by greater follow up and supervision [13, 22–24].

The variation of the mean scores for trained and untrained HCWs was from 40% to less than 60% unlike for the performance of HCWs trained in the Integrated Management of Childhood Illness in Benin which ranged from 15 to 88% [25]. This may be accounted for by the differences in the assessment approach.

Male HCWs performed better than their female counterparts. The same conclusion was made using 50 MCQs elsewhere [26]. The male gender was associated with retention of knowledge unlike in cardiopulmonary resuscitation and community maternal and newborn health training where there was no gender association with retention of knowledge [27, 28]. Faith-based health facilities were negatively associated with retention of knowledge as compared to state-owed health facilities. This may be due to lack of motivation probably because of less competitive compensation packages, and unclear career development in Faith-based health facilities [14]. Being a permanent staff member was associated with retention of knowledge. This may be due to psychological boast and assurance of a monthly income as compared to HCWs with temporary status. HCWs working in rural settings as compared to those in urban areas were associated with higher odds for retention of knowledge. This may be accounted for by the fact that rural areas might have less workload and the HCWs had more time to response to survey questionnaires with more concentration. Longevity at PMTCT of HIV, MNCH and Paediatric services was positively associated with retention of knowledge. Doing the same thing over and over may lead to a better understanding and retention of the knowledge. On-site training of other colleagues was associated with retention of knowledge. This may be due to prior preparation before the training of the colleagues which served as revision and permitted the trained HCWs to master the guidelines and concepts of PMTCT of HIV.

Univariate logistic regression showed association between retention of knowledge and service but not in the multivariable logistic analysis as corroborated by the study on cardiopulmonary resuscitation and service (working at accident and emergency departments) [27]. The difference may be due to methodological variations and outcomes being assessed. HCWs who spent longer duration in the same service understood better the guidelines, and polices and therefore performed better [10].

There was no association for retention of knowledge and grade of HCWs. Also, nurses performed nearly as well as doctors at 18 and 24 months after training on PMTCT of HIV. This was the same observation elsewhere [13, 27]. There was no association between age, and marital status with retention of knowledge as observed in community maternal and new-born health training [28], though contrary to the observation of an effect between age and knowledge in cardiopulmonary resuscitation [27]. Regression analysis showed no association between the level of education and retention of knowledge. This is contrary to community maternal and new-born health training post-test scores for Prevent Problems Before Baby Is Born at 18 months which showed an association [28].

We are not aware of any studies that assessed retention of trained HCWs on PMTCT of HIV services, retention of knowledge over time and correlates of retention of knowledge on PMTCT of HIV by HCWs. The current study shows a positive impact of training on the improvement of HCWs’ retention of knowledge but observed a progressive attrition of knowledge over a 3 year period. This translates into a 0.9% knowledge attrition for 67.9 to 64% of the HCWs from 1st to 2nd year and 2.2% knowledge attrition for 64 to 58.3% of the HCWs from 2nd year to 3rd year. Using a parsimonious extrapolating to the 4th year, 52.6% HCWs would have a mean score of 50.4% and in the 5th year 46.9% of the HCWs would have a mean score of 49.1% for retention of knowledge. Based on our findings we recommend training of HCWs working in PMTCT of HIV, MNCH and Paediatric services during the implementation of PMTCT programmes. Using a 50% threshold for retention of knowledge, we recommend re-training 4 years after initial training in order to maintain the knowledge of HCWs above the average score and render effective PMTCT of HIV programmes. Age, grade, years of work and education attainment may not enhance retention of knowledge for HCWs working at PMTCT of HIV, MNCH and Paediatric services. However, training programmes for HCWs of PMTCT of HIV may be made more effective by taking into consideration gender, type and location of health facility, employment status, longevity at PMTCT of HIV services and training of other colleagues on-site after initial training. PMTCT of HIV is a multi-prong complex intervention involving many actors and policies with many operational challenges having a threefold objective; combating HIV/AIDS, reducing child mortality and improving maternal health. We investigated a component that is rarely researched but in need of consistent, comprehensive and coherent data for making decisions. Accordingly, we recommend more research to provide evidenced-based data and enhanced health policies tailored to address declining knowledge of HCWs after training for the strengthening of health services and health systems in under-resourced settings.

## Conclusion

This study revealed high retention of HCWs in PMTCT of HIV, MNCH and Paediatric services, average retention of knowledge by HCWs and knowledge attrition overtime following training. Correlates of knowledge included: gender, type of health facility, employment status, location, longevity at PMTCT services, and training other colleagues. The age, grade, level of education and years of work were not associated with retention of knowledge. To ensure sustainability of mother–baby dyad re–training of HCWs after 4 years of initial training is recommended.

### Strengths and limitations

A number of strategies have been used by health care training programs to assess and ascertain changes in health providers’ skills and knowledge. Assessments have entailed pre- and post- tests knowledge [10], observation of skills by trainers [11], simulated clinical examinations [12], health provider self-reports [12], interviews with end users who are the recipients of the care [11], and post-training evaluations to assess retention of knowledge and skills over time [10, 12]. Different methods with different educational implications which may not give similar results are used to assess retention of knowledge. In educational settings, open-ended questions (cued recall) and true-false questions (recognition), relearning and transfer retention are used. For this study we used multiple choice questions (MCQs) with the added advantage that it uses a mixture of recall and recognition methods [29]. Our large sample size did not warrant the masking of any confounding relationship between retention of knowledge and dependent variables. This study assessed retention of knowledge by trained HCWs over a period of 3 years who underwent training by the PEPFAR team and compared it with untrained HCWs working at the PMTCT of HIV, MNCH and Paediatric services.

We did not assess motivation factors which considerably influence retention over time [30]. Retention of knowledge may also depend on supervisory visits and the importance accorded to the implementation of the programme by the health system which was not ascertained. We did not include the number of trained health workers at the health facility in the models. This is thought to be associated with case management [31] which could influence retention of knowledge. We did not compare our results with post-training scores.

## Abbreviations

AIDS: Acquired Immune Deficiency Syndrome
ANC: Antenatal Care
ANOVA: Analysis of Variance
AOR: Adjusted Odds Ratio
ARV: Anti-Retro Viral
CI: Confidence Interval
DFH: Department of Family Health
DHS: District Health Services
DHs: District Hospitals
EmONC: Emergency Obstetrics and Neonatal Care
FP: Family Planning
GHM: Global Health Metrics
HCs: Health Centres
HCW: Health Care Worker
HIV: Human Immunodeficiency Virus
IHCs: Integrated Health Centres
MCQ: Multiple Choice Question
MDGs: Millennium Development Goals
MNCH: Maternal, Neonatal and Child Health
MoH: Ministry of Health
MTCT: Mother-to-Child Transmission
OR: Odds Ratio
PEPFAR: President’s Emergency Plan for AIDS Relief
PMTCT: Prevention of Mother-to-Child Transmission
SD: Standard Deviation
SDGs: Sustainable Development Goals
SDHs: Sub-Divisional Hospitals
SRH: Sexual and Reproductive Health
SSA: Sub-Saharan Africa
UOR: Unadjusted Odds Ratio
WHO: World Health Organisation
